# Controlling the Porosity and Biocidal Properties of the Chitosan-Hyaluronate Matrix Hydrogel Nanocomposites by the Addition of 2D Ti_3_C_2_T_x_ MXene

**DOI:** 10.3390/ma13204587

**Published:** 2020-10-15

**Authors:** Anita Rozmysłowska-Wojciechowska, Ewa Karwowska, Michał Gloc, Jarosław Woźniak, Mateusz Petrus, Bartłomiej Przybyszewski, Tomasz Wojciechowski, Agnieszka M. Jastrzębska

**Affiliations:** 1 Faculty of Materials Science and Engineering, Warsaw University of Technology, Woloska 141, 02-507 Warsaw, Poland; Michal.Gloc.WIM@pw.edu.pl (M.G.); jaroslaw.wozniak@pw.edu.pl (J.W.); mateusz.petrus.dokt@pw.edu.pl (M.P.); bartlomiej.przybyszewski.dokt@pw.edu.pl (B.P.); agsolgala@gmail.com (A.M.J.); 2 Faculty of Building Services, Hydro and Environmental Engineering, Warsaw University of Technology, Nowowiejska 20, 00-653 Warsaw, Poland; ewa.karwowska@pw.edu.pl; 3 Faculty of Chemistry, Warsaw University of Technology, Noakowskiego 3, 00-664 Warsaw, Poland; twojciechowski@ch.pw.edu.pl

**Keywords:** MXenes, chitosan, hyaluronate, nanocomposite hydrogel, antibacterial, porosity, computed tomography

## Abstract

A recent discovery of the unique biological properties of two-dimensional transition metal carbides (MXenes) resulted in intensive research on their application in various biotechnological areas, including polymeric nanocomposite systems. However, the true potential of MXene as an additive to bioactive natural porous composite structures has yet to be fully explored. Here, we report that the addition of 2D Ti_3_C_2_T_x_ MXene by reducing the porosity of the chitosan-hyaluronate matrix nanocomposite structures, stabilized by vitamin C, maintains their desired antibacterial properties. This was confirmed by micro computed tomography (micro-CT) visualization which enables insight into the porous structure of nanocomposites. It was also found that given large porosity of the nanocomposite a small amount of MXene (1–5 wt.%) was effective against gram-negative *Escherichia coli,* gram-positive *Staphylococcus aureus,* and *Bacillus* sp. bacteria in a hydrogel system. Such an approach unequivocally advances the future design approaches of modern wound healing dressing materials with the addition of MXenes.

## 1. Introduction

Chitosan is a linear copolymer of β-(1–4) linked 2-acetamido-2-deoxy-β-D-glucopyranose and 2-amino-2-deoxy-β-D-glycopyranose. It one of the most common natural polymers obtained by alkaline deacetylation of chitin, which occurs in the skeletons of various invertebrates, such as mollusks and arthropods. It is also present in the cell wall of algae and fungi [1]. Chitosan is well-soluble and stable in an acidic environment, but insoluble in a neutral environment [2]. It is cationic which makes it unique among other polysaccharides and specific in the context of biological properties [3]. Thanks to its biocompatibility, it can be used medicinally in the area of implantation [4], wound healing promotion [5], and exhibits bacteriostatic effects [2,6,7]. Due to the positive charge at physiological pH, chitosan is also bioadhesive; this increases retention at the site of application [8]. Since chitosan is metabolized by human enzymes e.g., lysozyme, it can be considered also as a biodegradable material [9]. It also shows a variety of biological effects such as antifungal [10] and plant defense stimulation [11].

Chitosan, thanks to its hydrophilicity, functional amino groups or cationic charge, has been commonly used as a component of hydrogels, i.e., materials with high affinity for water, the dissolution of which is impossible due to chemical or physical bonds formed between polymer chains [12]. Using chitosan, hydrogels can be implemented in the intelligent delivery of macromolecular compounds such as peptides, proteins, antigens, or genes [3,13]. As can be seen, chitosan matrix composites have many positive features such as completely natural origin and excellent biocompatibility [14]. On the other hand, they are characterized by a specific porosity which is difficult to control [15]. Several techniques have been reported in the literature to control the pore size distribution of chitosan hydrogels. Salt leaching uses different salts or sugar particle sizes to determine pore sizes but requires the use of organic solvents that affect the properties of the final product [16]. The high pressure CO_2_ over an aqueous polymer solution with a crosslinker leads to only reduction of the fraction of large pores [17]. Currently, the feasible freeze-drying techniques are being extensively developed, but are also challenging, because the pore size in the final structure is influenced by many hardy-controllable factors [18,19,20] One feasible approaches is the addition of nanomaterials, including two-dimensional (2D) structures.

**Figure 1:**
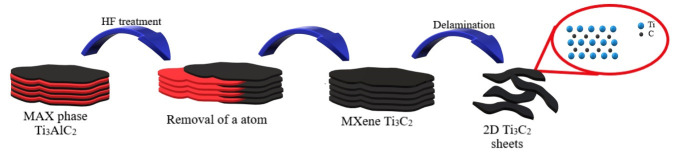
Schematic diagram of the preparation of 2D Ti_3_C_2_T_x_ MXene from starting Ti_3_AlC_2_ MAX phase.

MXene phases are new materials characterized by a 2D structure first obtained in 2011 [21]. The term ‘MXene’ reflects the stoichiometry of the material i.e., M_n+1_X_n_T_x_, in which M is a Group 3-7 transition metal, X corresponds to carbon or nitrogen, n = 1, 2, 3 or 4 [22], and T_x_ collectively refers to surface chemical groups such as -F, =O, and -OH. The synthetic method is based on the acidic etching of the initial M_n+1_AX_n_ phase [21,23], as a result of which the A atom—a group 13 or 14 metal is removed from the MAX phase structure. In this reaction the M_n+1_X_n_T_x_ system is formed. Among the numerous and diverse family of 2D materials, they are distinguished by the unique layered 2D structure of flakes which result in a sandwich-type arrangement of metal and non-metal layers arranged alternately. The process of obtaining delaminated 2D structure of the MXene phases is presented in Figure 1.

The presence of surface chemical groups such as -F, =O, and -OH is unique and characteristic for the MXenes family. It gives them a highly hydrophilic nature and allows for easy dispersion in various types of solvents [24,25], rising potential in hydrogel compositions [26,27,28] as well as interesting reversal adsorption properties for biomacromolecules [8,9]. A recent work [29,30] investigated adsorption of proteins with a positive surface charge, such as lysozyme or collagen. The surface of MXenes in an appropriate environment (pH change) resulted in their desorption. This is a significant advantage in surface reactivity over, for example, graphene, which is characterized by hydrophobic surface properties, and interactions that are not strong enough to provide good blending with precursor toward effective nanocomposite structures [31]. 

To date, several studies also indicated that MXenes have different bioactive features [32]. In 2016, Rasool et al. investigated the antimicrobial properties of the Ti_3_C_2_T_x_ MXene phase [33]. Studies have shown that this material showed higher biocidal activity against *B. subtilis* compared to *E. coli* at low nanomaterial concentrations, due to the interaction of nanomaterials with different cell wall structures of both strains. Jastrzębska et al. [34] compared the antibacterial properties of the two MXenes phases Ti_2_C and Ti_3_C_2_ against *E. coli* bacteria and found that the Ti_2_C phase did not affect the viability of the bacteria, while the Ti_3_C_2_ phase showed antimicrobial activity. Arabi Shamsabadi et al. [35] investigated the antimicrobial properties of colloidal Ti_3_C_2_T_x_ MXene nanoflakes against *B. subtilis* and *E. coli*, and foundthe antibacterial properties of Ti_3_C_2_T_x_ MXene nanoflakes depended on their size and exposure time. Because the Ti_3_C_2_T_x_ phase has a negative surface charge [36] it can potentially bind to the bacterial cell wall to damage the cell membrane and cell death [33]. The MXenes phases as biocides can be applied in filtration membranes [37,38,39], adsorptive removal of bacterial cells from water [32], or polymer matrix composites [40,41,42]. Composites based on synthetic polylactide were also obtained by electrospinning and they exhibited good antibacterial properties [43]. Also, due to their poor oxidation stability, different techniques were applied to stabilize MXenes e.g., addition to L-ascorbic acid (vitamin C) [44] or polyanionic salts [45]. To the best of our knowledge, these advances were not yet combined to obtain bioactive composite structures. 

Taken together, data indicate the promising influence of MXenes as an additive to nanocomposite structures with bioactive properties. However, the rationale behind the usage of MXenes in polymer matrix composites was based on only increasing the content until the accepted biocidal effect was obtained [39,46]. Apart from the above, still, not enough attention has been given to fully understand how the physicochemical and especially biological properties of such composites may change. It has not been clarified whether the use of MXenes makes it possible to obtain assumed porosity of composites based on natural polymer matrices such as chitosan or even mixed compositions with the addition of sodium hyaluronate and L-ascorbic acid. 

Therefore, the purpose of this work was to demonstrate the effectiveness of 2D Ti_3_C_2_T_x_ MXene in porous chitosan-hyaluronate matrix nanocomposites and the resulting hydrogels, stabilized by L-ascorbic acid addition. Strikingly, we observed that the addition of 2D Ti_3_C_2_T_x_ MXene to the nanocomposite structure adjusted the biocidal properties of the formed hydrogel. Moreover, it significantly decreased the nanocomposite porosity measured by the micro-CT technique. Altogether, our findings indicate that the usage of MXene 2D flakes in chitosan-based nanocomposites increases their application potential, by enabling porosity control of the developed structures while maintaining their antibacterial properties. Based on these findings, we envisage that the MXene materials will make the application of bioactive wound healing dressing materials more pronounced.

## 2. Materials and Methods

### 2.1. Preparation of the MAX Phase and MXenes

The Ti_3_AlC_2_ MAX phase was obtained using the Spark Plasma Sintering (SPS) technique, which was described in our previous work [47]. Briefly, the method comprises blending titanium, aluminum and synthetic graphite powders in a ball-type mill in isopropyl alcohol with a molar ratio Ti:Al:C of 3:1:1.9. Then, sieving (# = 300 µm) and drying the powders are carried out. The unique construction of the graphite matrix allows usage of the SPS pressureless technique during the reactive synthesis process [48]. The formula describing a reaction that takes place during the synthesis is presented below:

$$3\;\mathrm{Ti}+\mathrm{Al}+2\;C\to {\;\mathrm{Ti}}_{3}{\mathrm{AlC}}_{2}$$

At 1300 °C and under a vacuum are applied for the MAX Ti_3_AlC_2_ phase synthesis process. The heating rate is 250 °C min^−1^. The next stage concerns cooling and grinding of the MAX phase using an automatic mortar grinder (Retsch KM100) as well as subsequent sieving (# = 300 µm).

In the next step, the Ti_3_AlC_2_ MAX phase is etched with 48% (*v*/*v*) HF hydrofluoric acid (Sigma Aldrich) in a ratio of 1 g of powder per 10 cm^3^ of the acid. The process is carried out by stirring the suspension at a speed of 250 rpm on a magnetic stirrer for 24 h. The sedimented Ti_3_C_2_T_x_ phase was washed four times with deionized water. Subsequently, obtained multilayered Ti_3_C_2_T_x_ MXene was dried overnight at room temperature and stored at 5 °C for further use.

The delamination process is carried out in such a way that the obtained MXenes phase is mixed on a magnetic stirrer with water containing tetramethylammonium hydroxide (TMAOH) in a ratio of 1 mg MXene/1 mL H_2_O/10 mg TMAOH for 24 h at room temperature. A solution of pH~10 is obtained which is subjected to mild sonication for 6 h. To adjust the pH before the final washing step, concentrated 38% HCl (Avantor) was added dropwise until pH reached 7. It is worth noting that this technique is also recommended by Mathis et al. [49]. The mixture was then washed and centrifuged (3500 rpm, 5 min per cycle). The stable colloidal system MXenes obtained in this way is collected using vacuum filtration, dispersed in deionized water, and subjected to a freeze-drying process for 24 h to obtain a powdered material.

### 2.2. Preparation of the Porous Chitosan-Hyaluronate Matrix Nanocomposites

The experimental approach with nanocomposite structures synthesis first comprised the preliminary optimization of the composition of a base mixture of chitosan (CH) and sodium hyaluronate (SHA). The optimization process is described in detail in Supporting Information (SI). The optimal chosen composition is CH/SHA = 2:1 wt.% ratio with 3 wt.% addition of L-ascorbic acid (LAA). We used this knowledge to subsequently obtain nanocomposites with increasing 2D Ti_3_C_2_T_x_ MXene content (1%, 5%, and 10% by weight of dry matter). The synthesis procedure involves ultrasound-assisted redispersion of 2D flakes in distilled and blending with a matrix mixture of CH/SHA using a magnetic stirrer (30 min). Next, the blended mixture is placed on Petri dishes at 0.2 cm high (see details in Supporting Information), freezing at −45 °C, and subsequent freeze-drying (Alpha 2-4 LD Plus, Martin Christ, Osterode am Harz, Germany). The freeze-drying process was carried out in a uniform mode for 24 h under 1 mbar pressure. The dried composites with the chitosan-hyaluronate structures are further labeled as CH/SHA. These samples were stored at ambient conditions for further studies.

### 2.3. Studies on the Morphology and Structure of MXene and Chitosan-Hyaluronate Structures

The morphology and structure of freeze-dried 2D Ti_3_C_2_T_x_ MXene flakes were analyzed using a scanning electron microscope (SEM, LEO 1530, Zeiss, Lake Buena Vista, FL, USA). The preparation involves placing samples on carbon tape, sputtering with a carbon layer and analysis at 5.0 kV of accelerating voltage. The redispersed 2D flakes were tested on the presence of the Tyndall effect, drop-casted on a copper mesh and subsequently analyzed using a transmission electron microscope (TEM, PHILIPS CM 20, Philips International B.V., Amsterdam, The Netherlands). Single flakes are observed with a high angle annular dark-field (HAADF) scanning transmission electron microscopy (STEM) imaging whereas cross-sectionally oriented observations are carried out with Fourier transformation (FFT) coupled with inverted Fourier transformation (IFFT) mode and further accompanied by band intensity pattern. The elemental composition of 2D flakes is also evaluated with Energy Dispersive X-Ray Spectroscopy (EDS) coupled with TEM (PHILIPS CM 20, Philips International B.V., Amsterdam, The Netherlands). Further analysis of the morphology of CH/SHA matrix composites was performed using SEM. The samples are placed onto sticky carbon tape and sputtered with a thin carbon layer before SEM observations (SEM S3500N, Hitachi, Chiyoda, Tokyo, Japan) at an accelerating voltage of 15.0 kV.

### 2.4. Porosity Measurements of the Chitosan-Hyaluronate Structures

To reveal the key features of the developed nanocomposite structures, such as the effect of the addition of the MXenes phase to the polymer matrix on the porosity of the tested material, we performed a micro computed tomography (CT) analysis using an Xradia 400CT microtomograph, Carl Zeiss, Jena, Germany. This technique is a type of spectroscopic technique that allows you to obtain layered images of the test object using X-rays. To determine the porosity of tested samples, CT cross-sections were used, with a computer image reconstruction kit. Each sample is cut into ~5 × 5 mm sections and fixed in a horizontal position to the table inside the device chamber. The measurement was carried out at the voltage of 100 kV, a power of 10 W and a current of 250 µA between the cathode and the anode of an X-ray (tungsten) lamp. The exposure time is set to 2.5 s, angular rotation step to 1° with 6 photos per degree of rotation, and a resolution of 1 µm of voxel size. Over 1000 photos were obtained over a 180-degree rotation because of a standard five-hour scanning process. After obtaining the data, image reconstruction was performed using the NRecon software program, Microphotonics Inc., Allentown, PA, USA. Appropriate modes necessary for image correction were then introduced which were post alignment, beam hardening, ring artifact correction, and smoothing. Two-dimensional visualization of samples was obtained using DataViewer software, Microphotonics Inc., Allentown, PA, USA, while three-dimensional visualization is carried out using CT Vol software, Microphotonics Inc., Allentown, PA, USA. The images obtained after the reconstruction process used for the quantitative analysis of the microstructure in the CTAn program, Microphotonics Inc., Allentown, USA. Obtaining a binary image and removing defects enables estimating the percentage of porosity. The resolution of the method was 8.81 µm.

### 2.5. Studies on the Chemical Composition of MXene and Chitosan-Hyaluronate Structures

The potential chemical bonds forming between 2D Ti_3_C_2_T_x_ MXene flakes were also studied using Fourier Transform Infrared (FT-IR) spectroscopy (Nicolet iS5, Thermo Electron, Waltham, MA, USA) equipped with an Attenuated Total Reflectance (ATR) accessory and diamond crystal. The great advantage of this technique is that the potential interactions between components in nanocomposite can be easily revealed and samples do not require additional preparation. For all measurements, the spectral resolution was 2 cm^−1^, and each spectrum represents an average of 60 scans. For data analysis, the OMNIC 9.8.372 (Thermo Fisher) firmware version 1.02 softwarewas used.

### 2.6. TGA Measurements

Thermogravimetric analysis (TGA) was performed for assessment of water adsorption capability as well as estimation of degradation onset, using TGAQ500 analyzer (TA Instruments, New Castle, IN, USA). The temperature range was 30–630 °C at a heating rate of 20 °C min^−1^. TGA analysis were carried out in an air atmosphere and a 70 mL min^−1^ flow rate. 

### 2.7. Antibacterial Properties of Hydrogels

Bactericidal activity was examined in liquid cultures of bacteria: gram-negative *Escherichia coli* and gram-positive *Staphylococcus aureus* and *Bacillus* sp. The applied microorganisms came from the private collections of the Department of Biology, Faculty of Building Services, Hydro and Environmental Engineering, Warsaw University of Technology. Porous nanocomposites with different level (1, 5, and 10 wt.%) of phase Ti_3_C_2_T_x_ MXene, were soaked in the culture of the bacterial strain in nutrient broth (Biocorp) which allowed composites to form hydrogels. The initial number of bacteria in the culture was between 10^5^ and 10^6^ CFU/mL. The cultures were incubated for 24 h at 26 ± 1 °C. Serial dilutions of the cultures were accomplished and the number of bacteria was determined using a spread plate method, using agar (Biocorp) as a culture medium. The plates were incubated for 48 h at 37 ± 1 °C (*E. coli* and *S. aureus*) or 26 ± 1 °C (*B. subtilis*). The antimicrobial activity of the tested samples was evaluated as the percentage decrease in the number of bacteria in the cultures in the presence of the tested composites.

## 3. Results and Discussion

**Figure 2:**
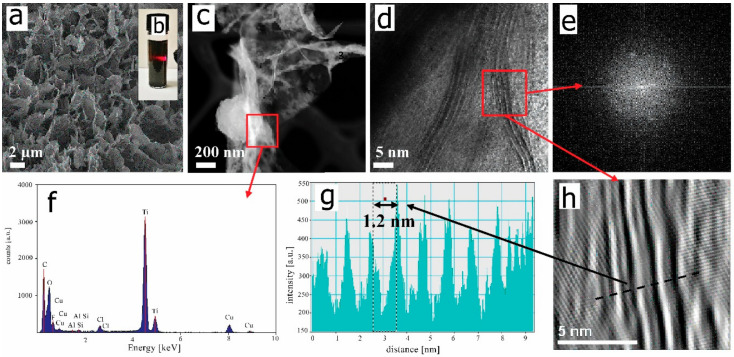
(**a**) SEM image of 2D Ti_3_C_2_T_x_ MXene flakes after freeze-drying. (**b**) Tyndall effect tested for redispersed nanocolloid. (**c**) STEM-HAADF and (**d**) HREM images of a single 2D flake after redispersion together with the corresponding (**e**) FFT image. (**f**) Results of the elemental EDS analysis. (**g**) Cross-sectional band intensity pattern. (**h**) IFFT image of the flake edge.

The present study concerns research on porous chitosan-hyaluronate matrix nanocomposites with the addition of 2D Ti_3_C_2_T_x_ MXene flakes as well as the resulting biocidal activity of the hydrogels. Before conducting biological tests, the nano-additives were thoroughly characterized. Analysis of the morphology and structure of 2D flakes of Ti_3_C_2_T_x_ MXene was carried out using SEMand as expected, the freeze-dried 2D Ti_3_C_2_T_x_ MXene flakes have irregular shapes with differently oriented edges (Figure 2a) and characteristic for this material [24,50]. These can be subsequently redispersed into a nanocolloidal solution, characterized by a presence of the Tyndall effect (Figure 2b), thereby suggesting the formation of a homogeneous aqueous dispersion of Ti_3_C_2_T_x_ MXene [51]. The separated micron-sized 2D flakes are visible on high angle annular dark-field (HAADF) scanning transmission electron microscopy (STEM) images (Figure 2c) and confirmed by our previous studies [52]. The high-resolution transmission electron microscopy (HREM) was taken from the randomly chosen sheet of the Ti_3_C_2_T_x_. Analysis reveals a characteristic layered structure of edges of 2D flakes (Figure 2d). Such multilayered structure was previously observed by Naguib et al. [24] The layered pattern is also confirmed by a fast Fourier Transform (FFT) imaging (Figure 2e) and extracted by the inverse fast Fourier Transform (IFFT) imaging which showed alternating layers of light and dark bands (Figure 2h). It should be noted that each bright band corresponds to a single Ti_3_C_2_ layer i.e., Ti-C-Ti-C-Ti. In contrast, each dark band corresponds to the spacing between the monolayers, formed after removal of an element from the MAX phase. To determine the characteristic distances between Ti_3_C_2_ monolayers in MXene flakes (light bands), the band intensity pattern was taken based on the corresponding IFFT image. The measured distance between maximal intensities of two Ti_3_C_2_ monolayers was 1.21 nm (Figure 2g) and is characteristic of the distance widening in flakes after delamination and weakening of bonds between monolayers as also confirmed by Lao et al. [53]. The FTT analysis also confirmed the hexagonal symmetry of the Ti_3_C_2_T_x_ [54]

We determined the elemental composition of 2D flakes using EDS analysis (Figure 2F). Those results indicated the presence of titanium and carbon as the two strongest signals (basic components of the MXene). There are also peaks from oxygen and fluorine, which are part of surface groups such as -F, -OH, =O [55], as well as minor amounts and chlorine due to the synthetic procedure with a pH adjustment as well as of copper from the sample preparation. The spectrum also shows small amounts of Al; these being incompletely etched Ti_3_AlC_2_ MAX phases.

Altogether, the results of flake characterizations indicated the successful synthesis and delamination into 2D Ti_3_C_2_T_x_ MXene that can be further used in a form of dry matter (lamellar structure after freeze-drying) for CH/SHA nanocomposites preparation. The delamination process of the etched MXene phase structure is carried out to obtain 2D flakes by breaking the weak out-of-plane van der Waals bonds between the layers. The 2D flakes obtained in this way, in contrast to the etched structure, formed a stable colloidal suspension in water [39,56]. Delamination allowed the use of the Ti_3_C_2_T_x_ phase in composites [57] with excellent functional parameters as electrodes for electrochemical capacitors or a “paper” with an exceptional volumetric capacity of four times higher compared to etched structure [56].

**Figure 3:**
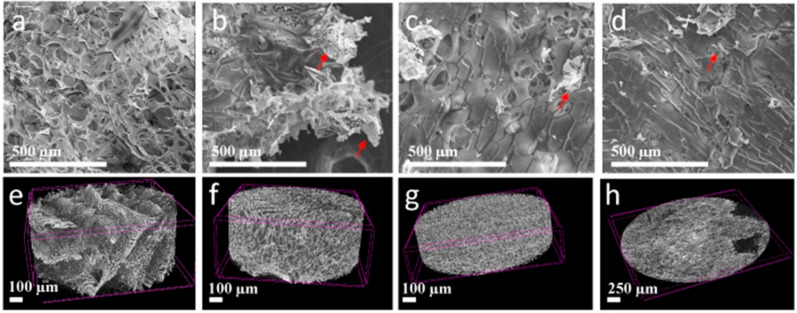
SEM and micro-CT images of the (**a**,**e**) reference CH/SHA matrix nanocomposite as well as modified with (**b**,**f**) 1 wt.%, (**c**,**g**) 5 wt.%, and (**d**,**h**) 10 wt.% of 2D Ti_3_C_2_T_x_ MXene flakes.

In the next step, the 2D Ti_3_C_2_T_x_ MXene flakes were used for the preparation of porous chitosan-hyaluronate matrix nanocomposites, stabilized by the addition of L-ascorbic acid. The freeze-dried nanocomposites were characterized before water addition and hydrogel formation. The SEM images of the reference CH/SHA matrix nanocomposite as well as modified with 1, 5, and 10 wt.% of 2D Ti_3_C_2_T_x_ MXene flakes are presented in Figure 3a–d. They revealed the combined and porous structure with macro and micropores characteristic of CH/SHA composites [58]. In Figure 3b–d the 2D flakes of the Ti_3_C_2_T_x_ MXene phase present in the polymer matrix are marked with red arrows. As seen in Figure 2b, most flakes of the Ti_3_C_2_T_x_ phase can be seen on the cut edges of the sample. As expected, they have irregular shapes. In Figure 3c,d MXene flakes are also present inside the polymer matrix. Due to the hydrophilic nature of the 2D Ti_3_C_2_T_x_ MXene flakes [59] they effectively blend with the chitosan-hyaluronate matrix. Therefore, they disperse throughout the entire volume of the composite. Moreover, by analyzing the Figure 3a–d pictures, it can be concluded that a decrease in the porosity of the tested samples is noted by increasing the levels of the MXene phase in the CH/SHA composites.

Porosity measurements were also carried out on dry nanocomposites using micro-CT (Computed Tomography) analysis. This technique was used because scanning electron microscopy (SEM) or gas adsorption does not provide clear information about the internal porous structure of the tested materials and whether it is homogeneous in the entire sample volume. The computed tomography method is commonly used to visualize the porous structure of materials. An additional advantage is that the tested materials are not damaged during the test and it provides its 3D structure. Carvalho et al. [60] used computed tomography to visualize the porous structure of chitosan - gelatin hydrogel hybrids. Also, Douglas et al. [61] successfully used this technique to check the homogeneity of chitosan hydrogels. Figure 3e–h shows a 3D reconstruction of reference CH/SHA as well as those modified with 1, 5 and 10% of 2D Ti_3_C_2_T_x_ MXene flakes. Asseen, the increased addition of 2D MXenes flakes causes a decrease in the height of the composite and decrease of its porosity. More details on the obtained images and porosity are presented in SI.

**Figure 4:**
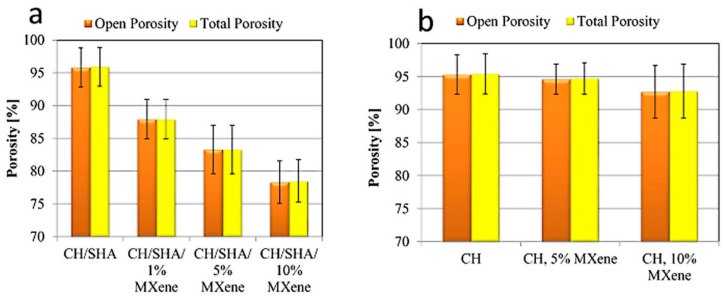
Results of total and open porosity calculations based on micro-CT data for the CH/SHA matrix nanocomposites modified with 1, 5, and 10 wt.% of 2D Ti_3_C_2_T_x_ MXene flakes (**a**). For comparison, results for CH composites (with no sodium hyaluronate) are shown in (**b**).

The results of open and total porosity, determined for individual samples, are presented in Figure 4. As shown, an increase of 2D Ti_3_C_2_T_x_ MXene flakes in the CH/SHA composite caused a significant decrease in open and total porosity, as shown in Figure 4a. The 1 wt.% addition resulted in a decrease in total porosity by ~8% compared to the reference sample. Further addition of 5 wt.% and 10 wt.% of the 2D MXene decreased porosity by ~13%, and ~18%, respectively.

Figure 4b compares the values of open and total porosity for chitosan composites (CH) without and with 5 and 10 wt.% addition of 2D Ti_3_C_2_T_x_ MXenes flakes. As can be seen the CH reference sample, without the addition of the MXene phase have a similar total porosity value compared to the composite containing SHA. It follows that the addition of SHA does not change the porous structure of the composite. The addition of 5 wt.% of MXenes phase causes a decrease in total porosity of CH by ~1%, while the addition of 10 wt.% causes a decrease in CH total porosity by ~3%.

**Figure 5:**
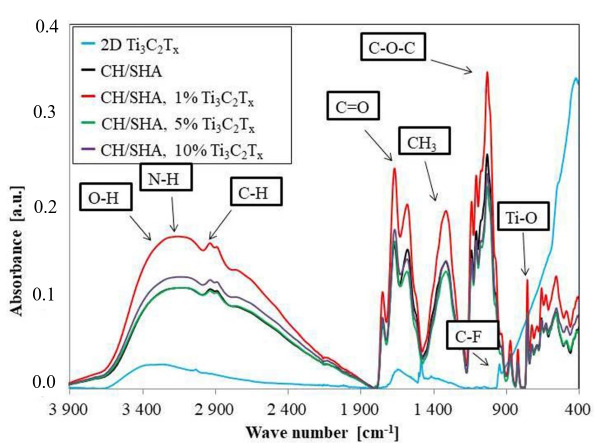
FTIR results for 2D Ti_3_C_2_T_x_ MXene flakes, reference CH/SHA matrix nanocomposite as well as modified with 1, 5, and 10 wt.% of the flakes.

The FTIR analysis was conducted on the pristine 2D Ti_3_C_2_T_x_ MXene (for comparison purpose) and dry nanocomposites with the addition of 1, 5, 10 wt.% 2D flakes of Ti_3_C_2_ MXene and presented in Figure 5. As shown in the spectrum of pristine Ti_3_C_2_T_x_ MXene a wide band at 3500–2900 cm^−1^ is observedand due to the stretching vibrations of hydroxyl groups –OH [62]. These are directly bonded to the titanium atoms on the surface of MXene, or come from water adsorbed on the surface. Peaks at 1320 cm^−1^ and 660 cm^−1^ are derived from O-H and Ti-O stretching vibrations as confirmed by Zhang et al. [63]. In the pristine MXene phase spectrum, a peak from the C-F group at 948 cm^-1^ can be seen and had been confirmed in our earlier work [47]. In the spectrum obtained for the polymer matrix without the addition of the Ti_3_C_2_T_x_ MXene sheets, signals from functional groups such as NH at 3176 cm^−1^ or CH at 2935 cm^−1^ overlap with the wide –OH at 3500–2900 cm^−1^. There are also signals from C = O at 1670 cm^−1^, CH_3_ at 1316 cm^−1^, and C-O-C at 1032 cm^−1^. This confirmed the presence of individual functional groups in the tested material. Analogous spectra were observed for CH/SHA composites modified with 2D Ti_3_C_2_T_x_ MXene. Hence, the expected signals are mainly of low intensity and overlap with the intense signals from the polymer matrix.

The small intensity on the Ti-O bonds (marked in Figure 5) clearly shows the beneficial effect of L-ascorbic acid (LAA) for both acquiring the appropriate pH for the CH polymer solubilization, but also for the stabilization of 2D Ti_3_C_2_T_x_ MXene. As reported previously [44], LAA, as a natural antioxidant, prevents surface oxidation of MXenes. Here, we also confirm that it prevents and minimizes the formation of the Ti-O bonds in the CH/SHA/Ti_3_C_2_T_x_ nanocomposite structure. Therefore, the problem with the oxidation of MXene was solved here by substituting acetic acid [64], widely used for CH preparation, with LAA. It is worth noting that LAA has a dual role in these composites. The first relates to lowering the pH value of the CH dispersion which allows for solubilization. The second is prevention of MXene oxidation. Herein, we take advantage of results reported by Zhao et al. [44] who discovered proved the highly beneficial effect of natural antioxidants on Ti_3_C_2_T_x_ MXene oxidation prevention. Therefore, the used MXene does not oxidize in the developed nanocomposite system.

Thermal analysis was used to determine the temperature of thermal degradation of these materials as well as to verify differences in material susceptibility to water. TGA data were obtained from the unmodified CH/SHA polymer matrix and modified with 1, 5 and 10 wt.% of 2D Ti_3_C_2_T_x_ MXene flakes. The charts are in the Supporting Information Figure S4.

Table 1 shows the temperatures of the first and second-largest weight loss. The first weight loss gives information regardingthe number of volatile residues in the polymer structure. In our case it is caused by the associated water evaporation. This confirms the high hydrophilicity of the structure [65]. As can be seen the lowest temperature, 59.03 °C was observed for the composite CH/SHA without addition of 2D Ti_3_C_2_T_x_ MXene flakes, while the highest temperature 63.73 °C, was observed for the composite with the 1% addition of MXene flakes. Comparing these results with the results of water absorption (Figure S5), it can be seen that the higher the temperature of the first weight loss corresponded to the greater the swelling capacity of the tested material. TGA results confirmed the higher water absorption and susceptibility to gelation of these materials.

The mechanism of gelation is self-assembly, as thoroughly described by Tontini et al. [66]. He noted that the disadvantage of MXenes was their low capability to gelation which appears as a reduction of the interconnectivity between 2D flakes. If the concentration of MXene in the freeze-drying mixture is too low, the MXene colloid collapses into a powdered form of aerogel-like porous flakes [67]. This problem was partially solved by using various additives such as ethylenediamine (EDA) [68], thiourea dioxide, ammonia [69], polyacrylic acid (PAA) [70], or divalent metal ions. The additives enhance the interconnectivity between material branches during gelation by simply filling the available space, inducing chemical cross-linking, chemical reduction or enhancing electrostatic interactions between the chemical groups of the mixed agents [66]. In this study, we took advantage of the electrostatic interactions between the mixed and then freeze-dried materials. It was important to optimize the concentrations of the components to achieve the optimal material parameters. It is worth noting that upon increasing the MXene concentration, the concentration of the chitosan was lowered together with lowering the optimal balance between the chemical groups available for electrostatic interactions. This highly influences the gelation process and porosity of the final freeze-dried structure. Therefore, the effect of reducing the porosity of the tested material is observed due to the increase of the Ti_3_C_2_T_x_ MXenes phase addition.

Table 1 shows the second weight loss which is related to the onset of degradation of the tested materials. The addition of the 2D Ti_3_C_2_T_x_ MXene flakes to CH/SHA composites does not significantly change their degradation temperatures, all of which are ~169 °C

Miranda et al. [71] analyzed TGA in their work on hyaluronic acid (HA), chitosan and composites with a weight ratio of CH/HA 1:1, 3:7 and 7:3. The results showed that HA degraded at 225 °C, CH around 285 °C, while composites degrade between 225 and 285 °C. Bazmandeh et al. [72] obtained similar results for pure chitosan. In their work, chitosan degraded at 250 °C. As can be seen in Table 1, the obtained composite structures degraded at a temperature of ~169 °C, which may be because sodium hyaluronate was used in the composites, not hyaluronic acid, as in the case of Miranda and Bazmandeh’s works.

Damaged skin secretes a body fluid that forms a sterile wound environment [73]. The damaged epidermis can also be a habitat for different microorganisms, including bacteria species that cause inflammation [74]. Therefore, an intelligent dressing should be an active, moist environment, facilitating rapid wound healing. It was noticed that the CH/SHA/Ti_3_C_2_T_x_ nanocomposite readily absorbed water and formed a hydrogel [71]. Therefore, we assumed that after immersion in a water-based environment, in which bacteria cells are present, it should effectively support the disinfection of the wound, facing the most important challenges in the healing process.

**Figure 6:**
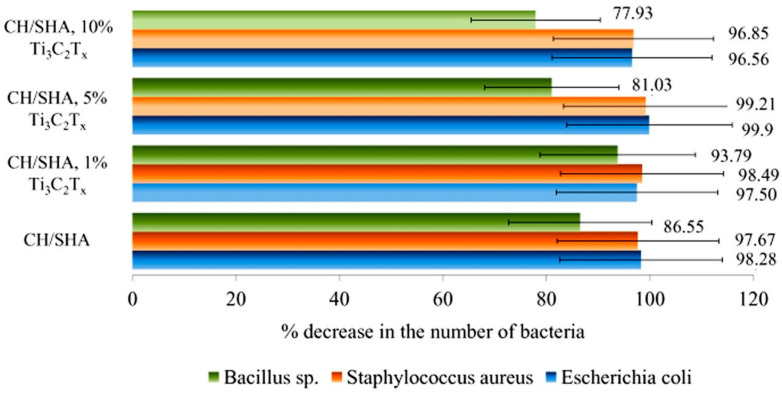
The effectiveness of the tested hydrogels in the elimination of bacteria in liquid culture.

Therefore, bactericidal activity was tested using three bacterial strains: gram-negative *Escherichia coli* and gram-positive *Staphylococcus aureus* and *Bacillus* sp. The tests were carried out on a reference CH/SHA sample and nanocomposites varying by the content of 2D Ti_3_C_2_T_x_ MXenes flakes (1, 5, and 10 wt.%), and those results are presented in Figure 6.

The results indicate that the addition of small amounts (1 or 5 wt.%) of the Ti_3_C_2_T_x_ phase to CH/SHA composites does not significantly influence the antibacterial properties of the nanocomposite, allowing for its maintenance at a desirable level.

It should be noted however that the differences in microbial susceptibility to the biocidal activity of nanocomposites can depend on the type of microorganisms. In the presence of a small amount of MXene (1 wt.%) the visible antibacterial effect (exceeding 90% of growth inhibition) was observed for all tested bacterial strains), while for the nanocomposite with 5 and 10 wt.% of MXene—only for *Staphylococcus aureus* and *Escherichia coli*.

The elimination of bacteria *Staphylococcus aureus*, which can be related to wound infections, exceeded 99% in this work, which is a promising result from a future practical application of this material. There is evidence that both chitosan and sodium hyaluronate are characterized by antibacterial properties [75,76]. The pure Ti_3_C_2_T_x_ phase (not surface-oxidized and not surface-modified) does not reveal the antimicrobial effect [47] because of the absence of the surface Ti_x_O_y_ oxides [52]. As can be seen, increasing levels of 2D Ti_3_C_2_T_x_ MXene, stabilized by L-ascorbic acid, did not significantly affect the biocidal properties of the chitosan-hyaluronate hydrogels. In the case of the material characterized by the large porosity, which is a key factor. Moreover, the low requirement for the 2D Ti_3_C_2_T_x_ MXene which should be added to the material allowed for successful optimization of the nanocomposite in this study.

## 4. Conclusions

This study presents chitosan-hyaluronate matrix hydrogel nanocomposites with the addition of 2D Ti_3_C_2_T_x_ MXene. It was revealed that addition of 2D Ti_3_C_2_T_x_ MXene lowered the porosity of the chitosan-hyaluronate matrix nanocomposite structures stabilized by vitamin C without the negative impact on its antibacterial properties.

The effect of the addition of the 2D Ti_3_C_2_T_x_ MXene on the structural properties of chitosan-sodium hyaluronate (CH/SHA) composites was examined using micro Computed Tomography analysis. Those results showed that the addition of 2D Ti_3_C_2_T_x_ MXene to the CH/SHA reduced the porosity of the obtained CH/SHA/Ti_3_C_2_T_x_ nanocomposites. The addition of the 2D MXene to a composite containing sodium hyaluronate caused a much greater decrease in the porosity of the tested material compared to composites not containing SHA.

FTIR analysis of the chitosan-sodium hyaluronate composites without and with 1, 5 and 10 wt.% 2D sheets of Ti_3_C_2_T_x_ MXene showed that there was no chemical reaction between the polymer matrix and the MXenes phase; therefore, no new chemical bonds were observed. Between both materials, only short- and long-range physical interactions occured, such as van der Waals interactions or hydrogen bonding. As a result, signals from both the polymer matrix and the MXene phase were observed in the CH/SHA/Ti_3_C_2_T_x_ composite. Any new, non-expected signals from other chemical bonds were not observed.

The effect of the addition of the MXene phase on the biocidal properties of composites against *Escherichia coli*, *Staphylococcus aureus*, and *Bacillus* sp. depended on the type of bacteria. In some cases the slightly increased antimicrobial effect of the 2D MXene–modified composites was observed as compared to the reference material; however it did not exceed several percent. Therefore, the bioactivity of the material can be maintained simultaneously with the development of the porosity which is influenced by the 2D MXene addition. This can have a significant impact on the effectiveness and safety of modern dressing materials obtained from these composites.

## Figures and Tables

**Table 1:** TGA summary by first and second weight loss for the CH/SHA matrix nanocomposites modified with 1, 5 and 10 wt.% of 2D Ti_3_C_2_T_x_ MXene flakes.

	First Weight Loss	Second Weight Loss
CH/SHA	59.03 °C	169.65 °C
CH/SHA, 1% Ti_3_C_2_T_x_	63.73 °C	169.06 °C
CH/SHA, 5% Ti_3_C_2_T_x_	63.14 °C	169.65 °C
CH/SHA, 10% Ti_3_C_2_T_x_	61.38 °C	169.06 °C

## Supplementary Materials

The following are available online at https://www.mdpi.com/1996-1944/13/20/4587/s1, Figure S1. SEM images of chitosan composites with 1 wt.% L-ascorbic acid (a), (b) and 5 wt.% L-ascorbic acid (c), (d), Figure S2. 3D reconstruction of samples with composition: CH, 3 wt.% LAA (a), CH, 3 wt.% LAA, 5 wt.% MXene (c), CH, 3 wt.% LAA, 10 wt.% MXene (e), examples of slices in the XY, YZ, XZ planes of samples with composition CH, 3 wt.% LAA (b), CH, 3 wt.% LAA, 5 wt.% MXene (d), CH, 3 wt.% LAA, 10 wt.% MXene (f), Figure S3. Examples of slices in the XY, YZ, XZ planes of samples with composition CH:SHA 2:1, 3 wt.% LAA (a) with the addition of the 1 wt.% (b), 5 wt.% (c), 10 wt.% (d) Ti¬3C2Tx MXene flakes, Figure S4. Results of thermogravimetric analysis for the CH/SHA matrix nanocomposites (a) modified with 1 wt.%; (b) 5 wt.%; (c) 10 wt.%; and (d) of 2D Ti3C2Tx MXene flakes, Figure S4. The swelling of the reference CH/SHA matrix nanocomposite as well as modified with 1, 5 and 10 wt.% of 2D Ti3C2Tx MXene flakes, Table S1: Optimization of composites composition, Table S2. Porosity values obtained on the basis of analysis in the CTAn program.
