# Supplementary material for: Controlling the Porosity and Biocidal Properties of the Chitosan-Hyaluronate Matrix Hydrogel Nanocomposites by the Addition of 2D Ti3C2Tx MXene
Source: Materials (Basel). 2020 Oct 15;13(20):4587. doi: 10.3390/ma13204587 (PMC7602632; doi:10.3390/ma13204587)
Supplement: Supplementary file 1 [file materials-13-04587-s001.pdf]

materials

Supporting Information

Controlling the Porosity and Biocidal Properties of the Chitosan-Hyaluronate Matrix Hydrogel Nanocomposites by The Addition of 2D $Ti_3C_2T_x$ MXene

1. Optimization of Composition of the Nanocomposite Structures

Stock solutions of sodium hyaluronate (SHA) at a concentration of 0.02% and chitosan (CH) at a concentration of 1% were prepared. Because chitosan dissolves only in an acidic environment, L-ascorbic acid (LAA) has been added to sterile water. To dissolve the ingredients, the solutions were mixed with a magnetic stirrer and heated to 37 °C. The ratio of chitosan to sodium hyaluronate and the content of LAA were optimized.

The composition of the prepared composites is given in Table S1.

Table S1. Optimization of composites composition.

Lp.	Ratio CH to SHA	L-ascorbic acid content [wt.%]
1.	2:1	1%
2.	1:1	1%
3.	1:2	1%
4.	1:0	1%
5.	2:1	3%
6.	1:1	3%
7.	1:2	3%
8.	1:0	3%
9.	2:1	5%
10.	1:1	5%
11.	1:2	5%
12.	1:0	5%

2. Preparation of Composites

The prepared samples were frozen at −45 °C, then dried using an ALPHA 2-4 LDplus freeze dryer, from CHRIST. The materials were dried for 24 hours at −20 °C under 1 mbar pressure. The best parameters were found in the composite composition in which the ratio of chitosan to sodium hyaluronate was 2:1 and the content of LAA was 3 wt.%. A smaller addition of sodium hyaluronate meant that the obtained composites were hard, and fragile, in the case of a larger addition, the composites were too delicate and gelified quickly. The addition of LAA was also optimized and the optimal choice was 3 wt.% because a larger addition of L-ascorbic acid caused an increase in the hardness and porosity of the tested material, and thus increased its fragility, in the case of a smaller addition, the composite obtained changed after one week from white to light brown.

The height of the liquid column for freeze-drying was also optimized concerning the thickness of the composites produced. Samples with previously selected optimal composition were made (ratio of CH to SHA 2:1 and addition of 3 wt.% LAA). Samples of thickness: 0.7 cm, 0.4 cm, and 0.2 cm were made. The optimal thickness was 0.2 cm because in the 0.7 cm and 0.4 cm thick the material was quite stiff and difficult to deform, which reduced its application properties.

3. Examination of Composite Morphology Using SEM

SEM analysis of chitosan composites with 1 and 5 wt.% of LAA content was performed at various magnifications. The results are presented in Figure S1.

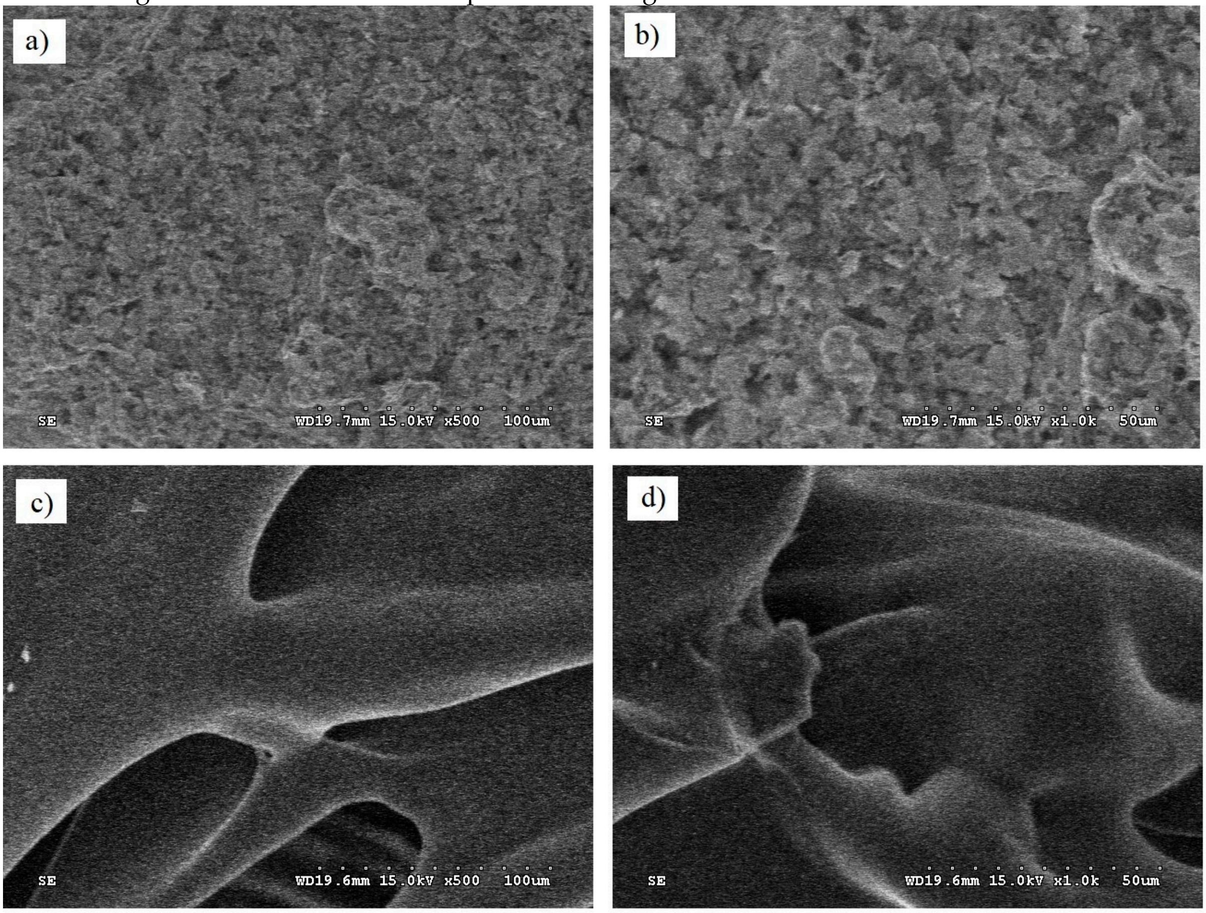

Figure S1. SEM images of chitosan composites with 1 wt.% L-ascorbic acid (**a**), (**b**) and 5 wt.% L-ascorbic acid (**c**), (**d**).

4. Computed Tomography Results

Micro CT (Computed Tomography) analysis was performed, samples composed of chitosan without the addition of MXenes phase, and with 5 and 10 wt.% addition of 2D $Ti_3C_2T_x$ MXene flakes. As a result of photo processing, 8-bit images were obtained, which allowed obtaining a three-dimensional tomographic model of the tested samples. The drawings show examples of slices of samples in the XY, YZ, XZ planes, and 3D reconstruction of individual materials.

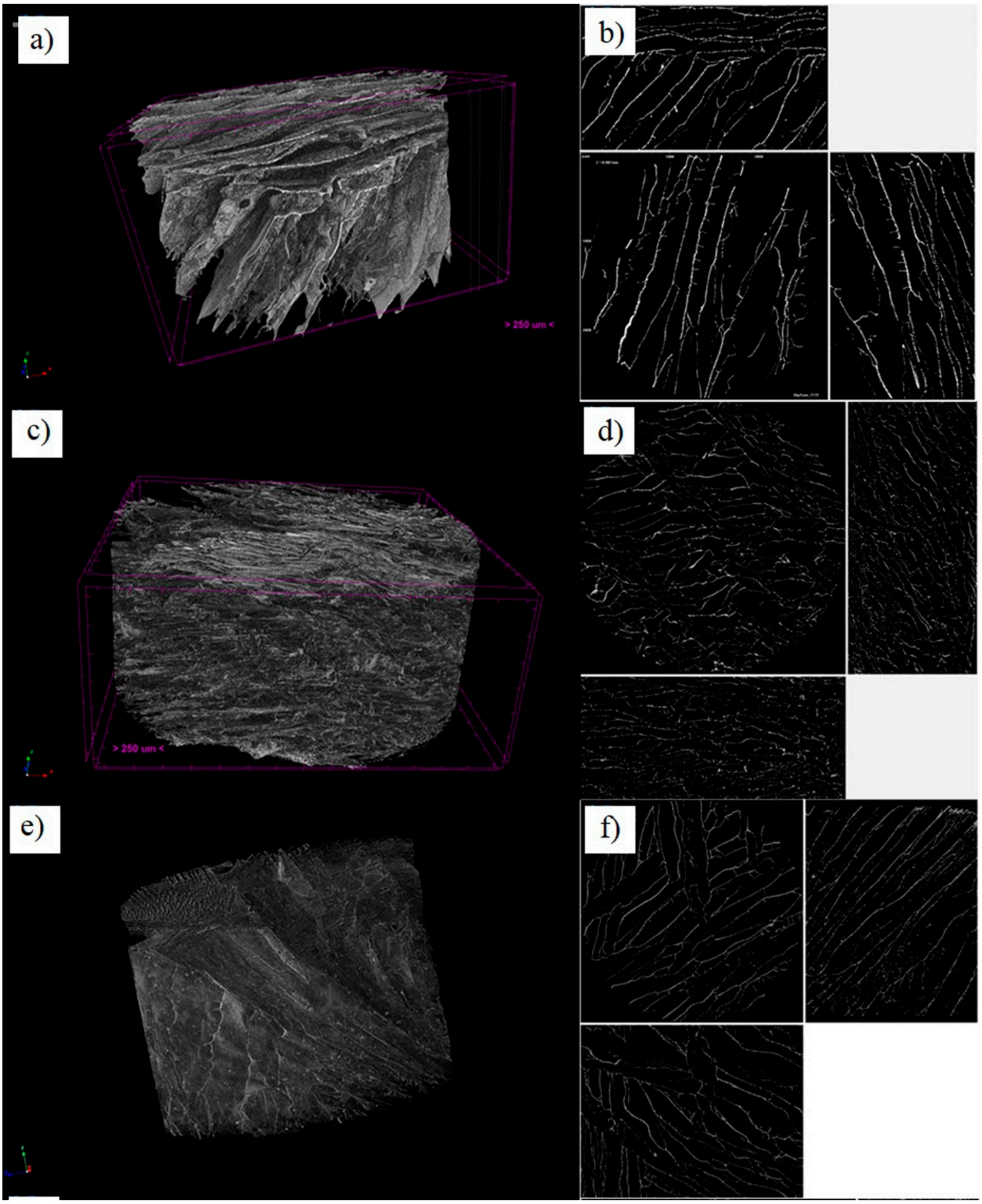

Figure S2. 3D reconstruction of samples with composition: CH, 3 wt.% LAA (**a**), CH, 3 wt.% LAA, 5 wt.% MXene (**c**), CH, 3 wt.% LAA, 10 wt.% MXene (**e**), examples of slices in the XY, YZ, XZ planes of samples with composition CH, 3 wt.% LAA (**b**), CH, 3 wt.% LAA, 5 wt.% MXene (**d**), CH, 3 wt.% LAA, 10 wt.% MXene (**f**).

Examples of slices in the XY, YZ, XZ planes of samples with composition CH:SHA 2:1, 3 wt.% LAA without and with addition of the 1, 5, 10 wt.% 2D $Ti_3C_2T_x$ MXene flakes are presented in Figure S3

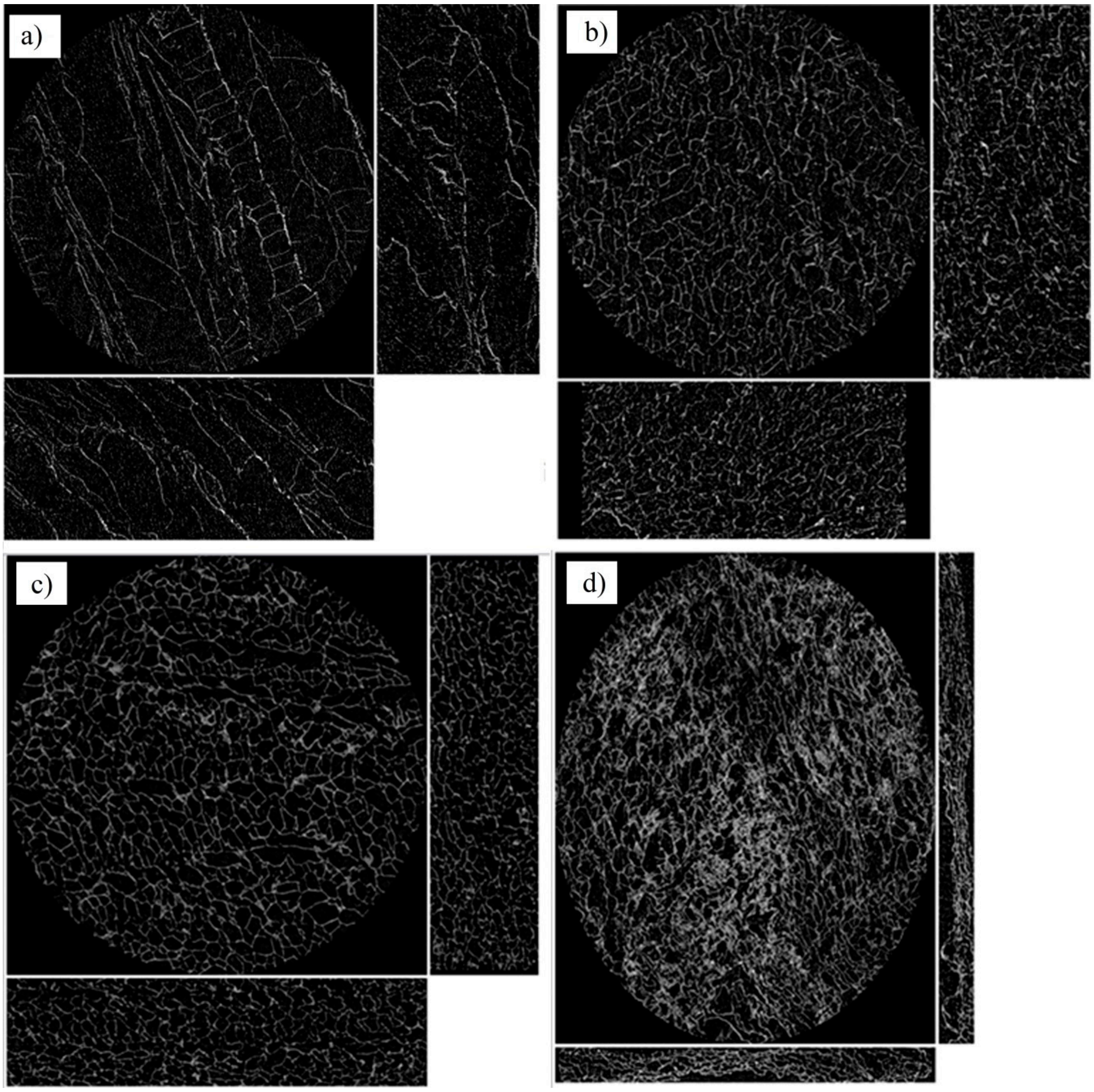

Figure S3. Examples of slices in the XY, YZ, XZ planes of samples with composition CH:SHA 2:1, 3 wt.% LAA (**a**) with the addition of the 1 wt.%; (**b**) 5 wt.%; (**c**) 10 wt.%; (**d**) $Ti_3C_2T_x$ MXene flakes**.**

The calculated values of closed, open and total porosity determined for individual samples are presented in Table S2.

Table S2. Porosity values obtained on the basis of analysis in the CTAn program.

Sample Name	Closed Porosity [%]	Open Porosity [%]	Total Porosity [%]
**CH, 3 wt.% LAA**	0, 4	95, 3	95, 4
**CH, 3 wt.% LAA, 5 wt.% MXene**	1, 2	94, 6	94, 7
**CH, 3 wt.% LAA, 10 wt.% MXene**	1, 1	92, 7	92, 8

Analyzing the above table, it can be concluded that the addition of the 2D $Ti_3C_2T_x$ MXene flakes causes a significant decrease in the closed porosity in chitosan-hyaluronate sodium composites. As the addition of MXene phase increases, a decrease in open porosity can also be observed.

5. Swelling experiment

The swelling properties of hydrogels correspond to their good ability to release contained in them macromolecules, e.g. drugs, Xu et al. [1] in their work, investigated the swelling capacity of

hydrogels with different weight ratios of chitosan to hyaluronic acid 1:1, 2:1 and 3:1. The results showed that hydrogels with a 2:1 ratio of chitosan to hyaluronic acid had the lowest swelling capacity compared to the 1:1 and 3:1 ratio composites, which may be due to the 2:1 hydrogel composition: 1 is close to the highest gel density which results in the smaller pore size of network structure preventing the water absorption. Kim et al. [2] on the other hand, proved in his work that the increase in the amount of chitosan in hydrogels in relation to hyaluronic acid increased the swelling capacity of the tested material.

In our work, we conducted swelling tests for composites with a CH/SHA composition in the ratio of 2:1 with different content of the MXenes phase. The swelling ratios (ESR) presented in Figure.S4, were obtained by weighing the initial and swollen samples. To measure the swelling ratio, previously weighted dry sampels (W_{dry}) were treated with deionized water. After 2 h the excess water was removed from the surface with filter paper and the swollen samples (W_{wet}) were weighted at room temperature. The swelling ratio was determined according to the equation.

$$ESR = (W_{wet} - W_{dry})/W_{dry}$$

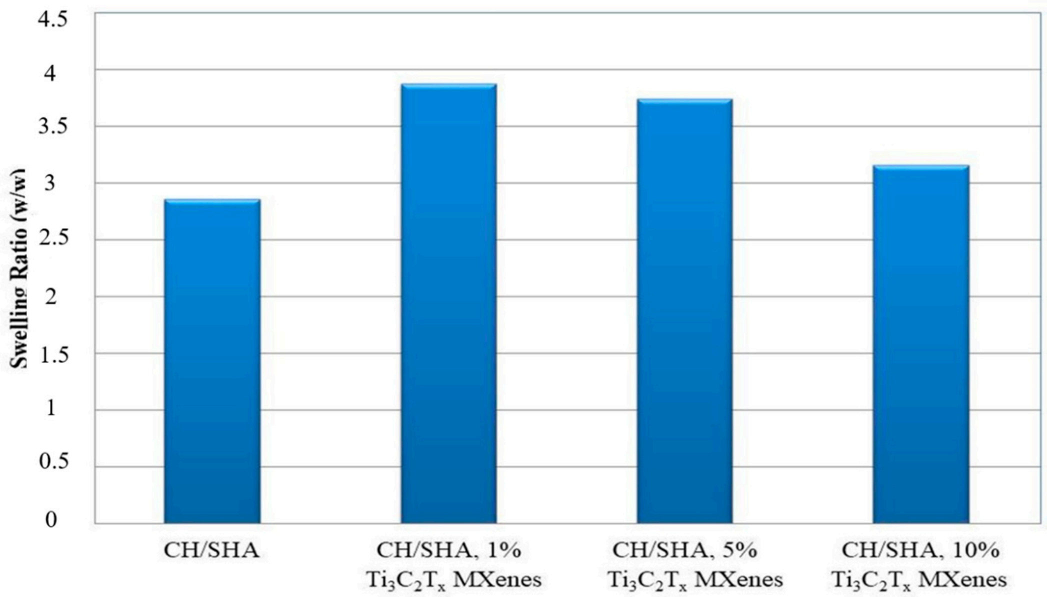

Figure S4. The swelling of the reference CH/SHA matrix nanocomposite as well as modified with 1, 5 and 10 wt.% of 2D $Ti_3C_2T_x$ MXene flakes.

As can be seen, the addition of the $Ti_3C_2T_x$ MXenes phase to the CH/SHA composite increases its swelling capacity. The highest swelling ratio is observed for composites with the addition of 1% by weight of $Ti_3C_2T_x$ MXenes phase. As the amount of MXenes phase additive increased, the swelling decreased.

References

[1]. Xu, H.; Zhang, L.; Bao, Y.; Yan, X.; Yin, Y.; Li, Y.; Wang, Z.; Huang, Z.; Xu, P. Preparation and characterization of injectable chitosan–hyaluronic acid hydrogels for nerve growth factor sustained release. *J. Bioact. Compat. Polym.*, **2016**, *32*(2), 146–162. doi:10.1177/0883911516662068.

[2]. Kim, S. J.; Lee, K. J.; Kim, S. I. Swelling behavior of polyelectrolyte complex hydrogels composed of chitosan and hyaluronic acid. *J. Appl. Polym. Sci.* **2004**, *93*(3), 1097–1101. doi:10.1002/app.20560.

© 2020 by the authors. Submitted for possible open access publication under the terms and conditions of the Creative Commons Attribution (CC BY) license (http://creativecommons.org/licenses/by/4.0/).
